# Observations on Catarrhal and Catarrho-Rheumatic Ophthalmia

**Published:** 1834-07-01

**Authors:** Frederick Tyrrell

**Affiliations:** Surgeon to the London Ophthalmic Infirmary.


### *Observations on Catarrhal and Catarrho-rheumatic Ophthalmia.*

By Frederick Tyrrell, Esq. Surgeon to the London Ophthalmic Infirmary.

Most frequently, during the spring and autumn, inflammation of the eye prevails for a short time as an epidemic, combined with catarrh, and therefore denominated catarrhal ophthalmia. This disease is usually very tractable, and requires but little medical skill to subdue it.

In the concluding part of the late winter, and from that period up to the present time, catarrhal ophthalmia has been more prevalent than I have known it during the last sixteen years, in which, as surgeon of the London Ophthalmic Infirmary, my attention has been especially directed to ophthalmic diseases. The disease has lately in numerous instances been unusually severe, and has affected the sclerotic as well a the conjunctiva, assuming a catarrho-rheumatic character. It is my intention, in the following paper, to give a description of the catarrhal and catarrho-rheumatic ophthalmia, and of the treatment which I have successfully pursued; and this I am induced to do, from having observed much difficulty among practitioners in the management of these diseases, arising from the want of a correct diagnosis, or from confounding the two diseases, or from considering them to be purely local.

### *Of Catarrhal Ophthalmia.*

It commences with stiffness and heaviness about the palpebræ, with slight irritation or itching of the ciliary margins, and a sensation as if some fine particles of dust had lodged on the surface of the globe : there is usually slight intolerance of light, with increased secretion of tears and agglutination of the lids during sleep. After a short time the patient experiences pain, which is increased by motion of the eyelids, or by exposure to bright light. These symptoms are aggravated about sunset.

On examining the eye there is perceived in the early stage slight thickening and redness at the free margins of the lids, and probably some coagulated secretion collected at the inner canthus, and about the cilia. The ocular portion of the conjunctiva exhibits a few tortuous vessels filled with red blood, whilst the palpebral portion is red, tumid, and villous, like crimson velvet; and in the lower folds between the ocular and palpebral parts of the membrane a little whitish or slightly yellow and thick mucous secretion is lodged.

The inflammation, however, soon spreads to the ocular conjunctiva, the vessels of which become extensively injected with red blood, and, on close inspection, are found to form a beautiful net-work. In severe cases the conjunctiva is partially thickened and elevated by deposition of serum between it and the sclerotic, constituting a partial chemosis, and occasionally small spots of ecchymosis are perceived from the rupture of some minute vessels.

If the disease extends farther it produces ulceration of the cornea.

This ophthalmic disease seldom exists without some symptoms of general catarrh, such as headach, sense of fulness about the frontal sinuses, sneezing, increased secretion from the nose, and some general febrile symptoms: the local and general diseases are usually reciprocal.

In young subjects the catarrhal-ophthalmia is often modified by a strumous diathesis, which occasions the local affection to be more severe, augments especially the intolerance of light and lachrymation, and disposes to the formation of pustules.

This disease may occur at any period of life. It is produced by a peculiar state of the atmosphere, during the continuance of which there is considerable difficulty in entirely removing the ophthalmia.

The treatment must be guided by several circumstances, as, the severity of the local diseases, the extent of constitutional disturbance, the condition of the general power, &c.

When there is simply affection of the palpebral conjunctiva with morbid mucous secretion and little general disturbance, and sufficient constitutional power, I prescribe a brisk aperient of calomel and colocynth, or calomel and rhubarb, or jalap, and afterwards a small quantity of some of the preparations of mercury with antimony, or with Dover's powder at night, and a mild saline aperient in the morning, also mild diet, principally vegetable and farinaceous, with quiet, and the avoidance of cold or damp, or of bright light.

Locally, I employ merely tepid water, to cleanse the eyes; or warm water to foment them when uneasy, and some simple ointment, which should be smeared upon the lids and eyelashes before the patient goes to sleep.

When the local disease is acute and the constitutional affection considerable, general or local bloodletting must be resorted to; the former being only necessary in persons of naturally full habit or subject to inflammatory diseases, and after bloodletting the treatment above described will be applicable.

In a few days the mild or acute form, if treated as I have described, passes into the chronic stage, which is denoted by a general mitigation of symptoms, but more especially by an alteration in the mucous secretion, which becomes thin and pale, and by the change in colour of the conjunctiva, which loses its brilliant aspect, and presents a more pale and lax appearance; at the same time it is found that the general power is failing under the continuance of the constitutional affection; the treatment must therefore be changed. Instead of exciting the secretions, they must now be kept as near as possible in their natural state, and a more generous diet must be allowed, but still the patient must abstain from such food as may tend to excite vascular action. It is necessary, in persons of feeble power, to give, in addition, some medicinal stimulus, as some of the preparations of bark, mineral acid, or ammonia.

To subdue the remains of the local disease, a slightly astringent lotion must be substituted for the tepid water, as, a solution of the acetate of lead half a grain, or a grain to the ounce; or a solution of alum a grain or two grains to the ounce; the sulphate or acetate of zinc a quarter or half a grain to the ounce, or nitrate of silver in the same proportion. Also a stimulating ointment must be applied instead of the milder form, as, the dilute citron ointment composed of half a scruple or a scuple of the unguentum hydrargyri nitratis, to two drachms of unguentum cetacei, or a scruple of the unguentum hydrargyri nitrico-oxydi to a drachm of the unguentum cetacei.

When catarrhal-ophthalmia is modified by a strumous diathesis, there is, as I have observed, intolerance of light in an increased degree, for the relief of which counter-irritation by means of blisters must be employed, in addition to the remedies already described; but the disease is always more obstinate when thus modified, and there is more difficulty in distinguishing between the acute and chronic form, inasmuch as the intolerance exists in both stages; the sufferings which it creates frequently induces the inexperienced surgeon to imagine that the disease must be acute.

As the constitution of strumous children is usually feeble, depletory measures must be adopted with caution, and the subsidence of the acute stage carefully watched, to prevent the unnecessary continuance of active treatment.

In producing counter-irritation, I am convinced that more good results from the repeated applications of small blisters, than from keeping the blistered surface open, or any other mode of inducing continued action. I direct a small blister to be placed either behind the ear, on the temple, or just above the eyebrow, and to be removed as soon as it has produced vesication; after which the part is to be dressed with some simple ointment, and allowed to heal: but, before it is perfectly well, I order another blister to be applied in the same way to another place; supposing the intolerance of light continues. I am confident also that in strumous children any cold applications to the eyes are injurious, and therefore I merely use tepid water in the acute stage, and depend in great measure on general remedies; and, in the chronic stage, if I employ stimulants, I have them applied warm.

When pustules are discovered on the ocular portion of the conjunctiva, there is not any necessity for deviation from the treatment which I have directed, although the eye is usually more irritable in consequence of the greater inequality of the conjunctival surface.

The above treatment commonly succeeds in subduing the general as well as the local disease; but now and then the acute local symptoms subside whilst the febrile action continues severe, and under these circumstances the surgeon should of course avoid the exhibition of tonics, and the use of local stimuli, as either would invariably occasion relapse.

The disease lately prevailing has been attended with considerable depression of nervous energy and diminution of general power, although the ophthalmia has in many instances been very severe; the acute stage has however readily yielded, and the principal difficulty has been in the management of the chronic form.

By far the greater number of cases in which I have been consulted have been in the chronic form, and in consequence of continued redness, pain, &c., treatment requisite for the acute form has been improperly continued; and the majority of these cases have rapidly recovered under the treatment directed for the chronic form of the disease.

*Of Catarrho-rheumatic Ophthalmia.*

This affection commences with symptoms similar to those of the catarrhal, but with more early suffering: for, in addition to the symptoms of the catarrhal disease, the patient experiences a constant dull aching pain in the globe of the eye, and in the temple and eyebrow: the globe feels tense, and as if it had been bruised; and these pains and sensations become so much augmented towards evening as to prevent sleep. The accession of pain is accompanied with intolerance of light and a feeling of dryness of the conjunctival surface, so that the motions of the eyelid upon the eye produces excessive pain; but occasional gushes of tears occur which for a few moments produce some relief: while the symptoms continue thus acute, the globe of the eye, the temple, and eyebrow, are extremely tender to the touch.

In the intervals between the severe attacks, when there is but slight intolerance of light, vision is frequently indistinct, and occasionally small black spots or muscæ are perceived by the patient.

On superficial inspection of the eye, the appearances denoting catarrhal-ophthalmia of an acute kind are apparent; such as the red palpebral margin, the thick mucous secretion, the thickened and villous state of the palpebral conjunctiva, with the tortuous vessels and vascular net-work on the ocular portion of the membrane; but an attentive examination soon discovers that the colour of the ocular conjunctiva is more uniform and deeper than in the simple catarrhal disease: and this upon further observation is found to depend upon the existence of another set of minute vessels filled with red blood, which can be traced in the intervals between the tortuous vessels of the conjunctiva as passing beneath them in a straight course from the margin of the cornea towards the orbit: these vessels are situated in the sclerotic coat; they are occasionally found more abundant in one part than another; as, for example, more upon the nasal than the temporal side of the cornea, and of course augmenting the depth of colour in such particular situations: it requires therefore a careful examination of the whole ocular surface to detect them.

When the vision is affected, being cloudy or troubled with muscæ, some change is perceptible in the iris; its pupillary aperture is contracted, its brilliancy is diminished, or its colour slightly altered; which circumstances are best detected by comparing the iris of the affected eye with that of the healthy organ: the motions of the affected iris also are sluggish. The iris suffers in consequence of inflammation extending to it from the sclerotic.

Sometimes a partial or complete ash-coloured line is seen between the cornea and vessels injected with red blood, most frequently the line forms a complete circle around the cornea; but I have seen it merely occupying a portion of the circumference of the cornea, being apparent at its temporal and nasal margins, and deficient superiorly and inferiorly. This appearance is considered by the continental and also many of our own ophthalmic surgeons, as a diagnostic of rheumatic inflammation. Such opinion, however, I consider to be erroneous, for I have seen a similar line present in iritis, simple sclerotitis, and choroiditis. I believe it results from the mode of junction between the sclerotic and cornea, occurring when any of the diseases alluded to are present, and seen most distinctly when the junction of the two structures is very oblique, but not at all evident when the textures are joined with little or no obliquity. Now and then the margin of the sclerotic overlaps or joins obliquely the circumference of the cornea, more in one part than another, and this I have observed most frequently on the temporal and nasal sides, so as to make the transverse diameter of the cornea less than the perpendicular diameter. When such formation exists and inflammation affecting the sclerotic occurs, the ash-coloured line is apparent only towards the nose and temple.

Commonly there is other evidence of rheumatic diathesis when this form of ophthalmia exists, the patient having pain about some of the joints or fibrous tissues in other parts of the body.

The exciting causes of the disease are cold and moisture; and I believe that the simple catarrhal affection is often converted into the catarrho-rheumatic, by too great a use of cold lotions.

*Treatment.* When inflammation preponderates in the conjunctiva, and the sclerotic is but slightly affected, the congestion of the vessels must be relieved by the application of a few leeches; at the same time I give the patient an active purgative, and then for a day or two I pursue the same plan as if the disease were simply catarrhal, excepting that when the circum-orbitar pains are severe, I direct blisters to be applied, or a few grains of the blue ointment with opium to be rubbed into the temple and forehead at night. Immediately the severity of the conjunctival affection has subsided, I prescribe a more nutritive diet, and small doses of bark and soda, sarsaparilla and lime-water, or some mild tonic with an alkali. I desire the patient to abstain from all acid food and fermented drinks.

Locally, warm water is occasionally used to cleanse the eye, but the patient must carefully wipe it, and not allow the surface of the lids to remain moist, as I am satisfied it augments the mischief.

The repetition of blisters is serviceable where there is much intolerance of light, or continual circum-orbitar pains.

When the eye continues irritable, with but little vascular congestion or symptoms of inflammation, I find considerable advantage from putting a drop or two of vinum opii into the eye, once or twice in the twenty-four hours. If this remedy produces pain, which is continued beyond eight or ten minutes, it does no good.

If the catarrhal affection of the conjunctive be slight, and the inflammation of the sclerotic severe, without however much affection of the iris, local bloodletting is rarely necessary. The patient is at first to be freely purged by some drastic medicine, after which a full dose of colchicum, with a narcotic, may be given, especially when there is any marked rheumatic diathesis: the best period for administering this dose is in the evening, a short time before the usual period of exacerbation. I have known it to subdue altogether the sclerotic inflammation, leaving but a slight catarrhal ophthalmia. If the first dose appears to alleviate the disease, it may be repeated every six or eight hours for three or four times; but I have never found any benefit from a longer continuance of this remedy, nor have I seen much advantage from it when it has occasioned disturbance of the stomach or bowels. I consider its beneficial action very uncertain.

Otherwise, I prescribe according to the state of general power, which it is of the utmost importance to observe, in order to treat successfully. Very often, when the local disease appears acute, the general power is extremely feeble; or, on the contrary, when the local disease is slight, the constitutional power is good; but occasionally, the general action is energetic when the local disease is severe. When the action of the heart and arteries evinces deficient power, although the local symptoms and appearances may indicate an acute form of disease, any active depletion likely to affect the system ought not to be adopted; blistering is the best means of relieving the local congestion; besides which, medicines should be given to correct any error in the principal secretions, especially those of the bowels, skin, and kidneys: the two latter I find most frequently to be deranged. Antimonials with salines, are therefore most serviceable after the bowels have been acted upon. The patient must be allowed a good nutritious diet, and even a small quantity of stimulus, if much addicted previously to its use.

As soon as the secretions are in a healthy condition, tonics should be exhibited, and if the general power is good I prescribe five grains of the pulvis cinchonæ, and an equal quantity of the sodæ subcarbonas exsiccata every four or six hours; and I know of no remedy so generally serviceable in the state of the disease above described. I am indebted to Mr. Wardrop for the suggestion of this medicine. I have frequently known it succeed in relieving inflammation of the sclerotic, when the larger doses of the same preparation have been taken some time without benefit. Should this remedy fail, I give the sursaparilla with lime-water, but with either medicine it is necessary to administer an occasional purgative, with or without some mercurial, according to the state of the biliary secretions.

When the patient's power is feeble, I usually commence with the sulphate of quinine, as a tonic, in doses of three or four grains every four or six hours; and, if there is a disposition to inordinate cutaneous secretion, I prescribe, in addition, twenty or thirty minims of the dilute sulphuric acid. Under these circumstances, also, a generous diet is requisite, and a moderate quantity of any stimulus to which the patient may have been accustomed. I have lately seen many cases in which the catarrho-rheumatic disease had continued, (principally however of a rheumatic character,) for six or seven weeks, or more; a depletory treatment having been persisted in, and in which a cure has been effected in as many days after the tonic plan has been adopted.

It sometimes happens that the disease has extended from the sclerotic to the iris before the patient applies for relief, or that the affection being treated under the supposition that it is merely a conjunctival inflammation, the remedies employed have not been sufficient to prevent the extension of the disease to the iris. Inflammation of the iris is indicated by a loss of brilliancy, an alteration in colour, a thickening of the pupillary margin, and a contracted and sometimes irregular condition of the pupillary aperture. When the iris is but slightly diseased, there is no occasion to deviate from the treatment which I have just described; but if the inflammation of this texture is decidedly developed, it is absolutely necessary to resort to mercurial treatment to stop its progress, otherwise the plan of depletion which I direct in the early stage of catarrhal or simple catarrho-rheumatic ophthalmia, may not arrest the inflammatory action in time to prevent deposit of lymph in the texture of the iris and in its pupillary margin, by which the pupil may be rendered permanently irregular, or its margin become in part adherent to the anterior capsule of the lens, and the transparency of the capsule itself be materially injured.

The exhibition of mercury must be carefully and guardedly resorted to, especially when the constitution is feeble, which I have observed it most frequently to be in persons suffering from this disease; nevertheless I deem the use of the remedy of the greatest importance, if given in small doses, at short intervals, whilst the patient is allowed a nutritious diet, and the secretions kept in good order; the surgeon may, by keeping a careful watch, always stop the exhibition of the medicine in time to prevent any mischief.

At the same time that mercury is given, the extract of belladonna should be applied to the eyebrow night and morning, to keep the pupil in a dilated state, and prevent any adhesions taking place between the pupillary margin of the iris and the anterior capsule of the lens, with a contracted pupil; or to aid in the separation of such adhesions, should they have been formed previously.

The mercurial treatment must be stopped as soon as the inflammation of the iris subsides, whether the system be affected by the remedy or not. I have seldom had occasion to give mercury so as to produce any marked effect on the system in these cases, and have always found that when the remedy has by accident, or inattention, created much general disturbance, the cure of the remaining disease in the sclerotic has been more difficult. The disease in the iris being subdued, the plan I have recommended for relieving the inflammation of the sclerotic should be immediately adopted.

In several instances which have come under my observation during the last few months, although the general and local diseases have not been very severe, the patients have experienced excessive debility, and have not recovered until they have had the benefit of change of air for a short time.

I have been desirous to shew, in the preceding observations, that it is of much importance, by careful examination of the organ and attention to symptoms, to ascertain the extent of disease before treatment be commenced, and that the treatment must be varied according to the extent and severity of the inflammation; but especially to point out to the existence of apparently severe local inflammation, with a feeble state of the constitution, which I have observed frequently during the late prevalence of these diseases; for I am satisfied that inattention to this circumstance has been the principal cause of want of success in the management of most of the cases which I have seen in consultation.

